# Is There Any Effect of the Physician Performing Embryo Transfer in IVF-ICSI Treatment: A Prospective Cohort Study

**DOI:** 10.1055/s-0041-1740473

**Published:** 2022-01-29

**Authors:** Zeynep Ozturk Inal, Hasan Ali Inal, Emine Aksoy, Sultan Mermer

**Affiliations:** 1 Department of Reproductive Endocrinology, In Vitro Fertilization Unit, Konya Training and Research Hospital, Konya, Turkey

**Keywords:** assisted reproductive technology, embryo transfer, infertility physicians, pregnancy rate, tecnologia reprodutiva assistida, transferência de embrião, médicos de infertilidade, taxa de gravidez

## Abstract

**Objective** To evaluate whether there is an effect of the physician who transfers the embryos on pregnancy rates in in vitro fertilization-intracytoplasmic sperm injection (IVF-ICSI) treatment.

**Methods** A total of 757 participants were analyzed between 2012 and 2017. Participants were classified according to 3 physicians who transferred the embryos: ([group 1 = 164 patients]; [group 2 = 233 patients]; [group 3 = 360 patients]). Baseline parameters and IVF-ICSI outcomes were compared between the groups.

**Results** No differences were determined between the groups regarding the baseline parameters (age, age subgroups [20–29, 30–39, and ≥ 40 years old)], body mass index (BMI), smoking status, infertility period, cause of infertility, baseline follicle stimulating hormone, luteinizing hormone, estradiol (E _2_ ), thyroid stimulating hormone, prolactin levels, antral follicle count, duration of stimulation, stimulation protocol, gonadotropin dose required, maximum E _2_ levels, progesterone levels, endometrial thickness on human chorionic gonadotropin (hCG) administration and transfer days ( *p* > 0.05). The numbers of oocytes retrieved, metaphase II (MII), 2 pronucleus (2PN), , transferred embryo, fertilization rate, day of embryo transfer, the catheter effect and embryo transfer technique, and clinical pregnancy rates (CPRs) were also comparable between the groups ( *p* > 0.05).

**Conclusion** Our data suggests that the physician who transfers the embryos has no impact on CPRs in patients who have undergone IVF-ICSI, but further studies with more participants are required to elucidate this situation.

## Resumo

**Objetivo** Avaliar se há ou não efeito do médico que realiza a transferência de embriões nas taxas de gravidez no tratamento com fertilização in vitro-injeção intracitoplasmática de espermatozoide (FIV-ICSI, na sigla em inglês).

**Métodos** Um total de 757 participantes foram analisados entre 2012 e 2017. Os participantes foram classificados de acordo com 3 médicos que transferiram os embriões: ([grupo 1 = 164 pacientes]; [grupo 2 = 233 pacientes]; [grupo 3 = 360 pacientes]). Parâmetros basais e resultados de FIV-ICSI foram comparados entre os grupos.

**Resultados** Nenhuma diferença foi determinada entre os grupos nos parâmetros basais (idade, subgrupos de idade [20-29, 30-39 e ≥ 40 anos)], índice de massa corporal (IMC), tabagismo, período de infertilidade, causa da infertilidade, hormônio folículo estimulante basal , hormônio luteinizante, estradiol (E2), hormônio estimulador da tireoide, níveis de prolactina, contagem de folículos antrais, duração da estimulação, protocolo de estimulação, dose de gonadotrofina necessária, níveis máximos de E2, níveis de progesterona e espessura endometrial na administração de hCG e nos dias de transferência ( *p* > 0,05). O número de oócitos recuperados, MII e 2PN, embrião transferido, taxa de fertilização, dia da transferência do embrião, o efeito do cateter e a técnica de transferência de embrião e taxas clínicas de gravidez (RCPs) também foram comparáveis entre os grupos ( *p* > 0,05).

**Conclusão** Nossos dados sugerem que o médico que transfere os embriões não tem impacto sobre as RCPs em pacientes que se submeteram a FIV-ICSI, mas mais estudos com mais participantes são necessários para elucidar esta situação.

## Introduction

Despite all of the developments in assisted reproductive technology (ART) since the first live birth following in vitro fertilization (IVF) in 1978, pregnancy rates have remained at between ∼ 35 and 45%. 1 2 3 4 5 In ART cycles, the method of embryo transfer (ET) is important for clinical pregnancy success in addition to features such as age, endometrial receptivity of the infertile woman, and embryo quality. 6 7 8 9 It has been claimed that faulty ET is responsible for between 25 and 30% of failed implantations, related either to the catheter application technique or to the experience of the clinician performing the ET procedure. 5 6 10 To minimize possible negative effects of different physicians on the clinical pregnancy rate (CPR) in ET, these procedures have been standardized by assisted reproduction clinics. Nevertheless, some studies have suggested that the physician who performs the ET may affect CPR success. 11 12 13 In the present study, we aimed to evaluate the effect of the physician who transfers the embryos on pregnancy rates in IVF-intracytoplasmic sperm injection (IVF-ICSI) treatment.

## Methods

### Study Participants and Data Collection

The present prospective study was performed at the Reproductive Endocrinology Department of the Ali Kemal Belviranli Maternal Women's Health and Children's Hospital. Outcomes of 757 fresh ICSI cycles were reviewed between January 2012 and December 2017. The inclusion criteria were participants aged between 20 and 44 years old, body mass index (BMI) between 18 and 35 kg/m ^2^ , regular menstrual cycles, no uterine abnormalities in the ultrasound, and normal baseline hormonal levels. Participants were excluded from the study if they were ≥ 45 years, with a BMI ≥ 35 kg/m ^2^ , or had any significant illness or metabolic disorders. Ethical board approval was granted from the institutional review board (2012/57). Written and oral informed consent was given from the participants. Data were obtained for age, BMI (kg/m ^2^ ), smoking status, infertility period, cause of infertility, baseline at day 3 for follicle-stimulating hormone (FSH), luteinizing hormone (LH), and estradiol (E _2_ ) levels, thyroid-stimulating hormone (TSH), prolactin, antral follicle count, stimulation parameters, IVF-ICSI outcomes, and CPR.

### Ovarian Stimulation and Oocyte Retrieval

Controlled ovulation stimulation was negotiated using the gonadotropin-releasing hormone agonist (GnRHa) or the flexible gonadotropin-releasing hormone antagonist (GnRHant) protocol.

### Embryo Transfer Procedure

Three senior physicians performed the ETs accompanied by ultrasonographic guidance (USG) (Logiq 200 Pro, General Electric, Seoul, South Korea) using an ET catheter system. A sterile speculum was introduced in the vagina in the lithotomy position, then the vagina and the cervix were cleaned using sterile cotton swabs. An embryologist loaded the embryos into a soft transfer catheter that was given to the ET physician, who deposited the embryos ∼ 10 mm from the uterine fundus under USG. The catheter was gently removed after 5 seconds. In cases of ET with external guidance, an initial catheter with inner sheath was inserted into the external cervical os, and then advanced through the cervical canal and the internal os to 10 mm of the uterine fundus under USG. The internal sheath was withdrawn, and a second catheter loaded with embryos was introduced in its place and advanced to ∼ 10 mm from the uterine fundus, where the embryos were deposited. Difficult transfers required the use of a stylet in addition to this form of external guidance. All catheters were immediately checked for retained embryos and blood, and the patient remained in the Trendelenburg position for ∼ 10 minutes. Patients who used the tenaculum were excluded from the study. Progesterone in the form of Crinone 8% gel (Serono, Istanbul, Turkey) at a daily dose of 90 mg for 14 days was given for luteal phase support. Baseline parameters and IVF-ICSI outcomes were compared between the groups. Clinical pregnancy was accepted as those with a gestational sac accompanying fetal heartbeat on ultrasound examination at between 4 and 5 weeks after the ET. The CPR was defined as the number of clinical pregnancy cycles/number of embryo transfer cycles × 100%. The number of embryos transferred (≤ 2 per patient) complied with the Turkish national regulations. The subjects were classified according to 3 physicians who transferred the embryos: ([group 1 = 164 patients]; [group 2 = 233 patients]; [group 3 = 360 patients]).

### Statistical Analysis

The statistical analyses were performed using SPSS for Windows, version 15.0 (SPSS Inc., Chicago, IL, USA). The Shapiro-Wilk test was used to examine the continuous variables with normal and non-normal distributions. The one-way analysis of variance (ANOVA) was preferred for the normally distributed continuous variables, while the Kruskal-Wallis test was used for the non-normally distributed continuous variables. Categorical data were analyzed by the Pearson chi-squared test, and the Fisher exact test was applied if the expected frequency was < 5 in > 20% of all cells. The continuous variables were presented as mean ± standard deviation (SD), and the categorical variables were presented as the number of cases and percentages. The Bonferroni adjustment was performed to control the type I errors for all possible multiple comparisons. A *p-* value < 0.05 was accepted as statistically significant.

## Results

**Fig. 1:**
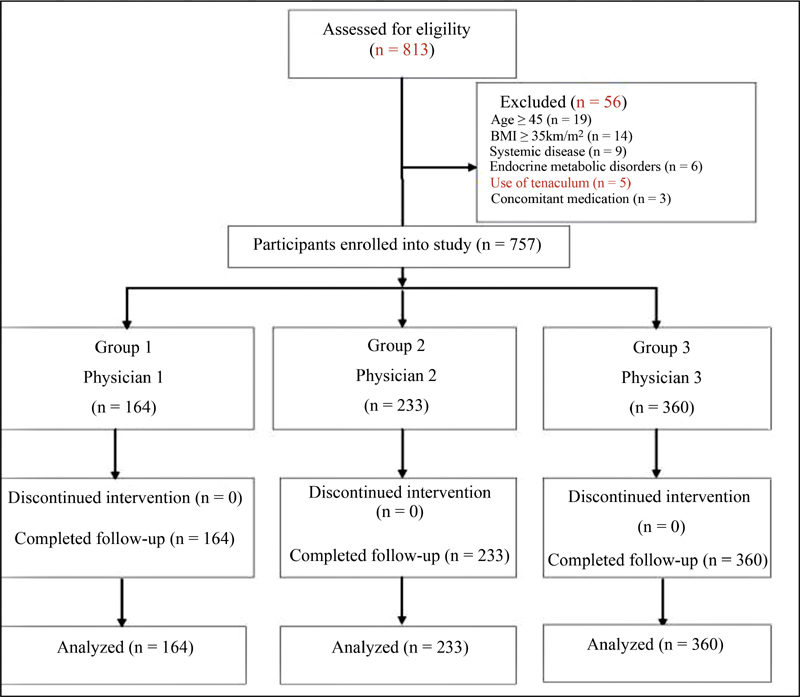
Enrollment and follow-up of the study subjects.

A total of 56 patients were excluded from the study, specifically those ≥ 45 years old ( *n* = 19), with BMI ≥ 35kg/m ^2^ ( *n* = 14), with systemic disease ( *n* = 9), endocrine or metabolic disorders ( *n* = 6), use of tenaculum ( *n* = 5), and concomitant medication ( *n* = 3). The remaining 757 participants were classified into the three ET groups and their outcomes were analyzed ( Fig. 1 ).

A comparison of the sociodemographic and stimulation characteristics of the participants is provided in Table 1 . There was no difference between the groups regarding age, age subgroups (20–29, 30–39, and ≥ 40 years old), BMI, smoking status, infertility period, cause of infertility, baseline FSH, LH, E _2_ , TSH, prolactin levels, antral follicle count, stimulation day, stimulation protocol, gonadotropin dose required, maximum E _2_ and progesterone levels, and endometrial thickness on hCG administration and transfer days ( *p* > 0.05).

**Table 1:** Demographic and stimulation characteristics of the patients

		Group 1 ( *n* = 164)	Group 2 ( *n* = 233)	Group 3 ( *n* = 360)	*p-value*
Age (years old)		30.31 ± 5.48	29.21 ± 4.54	29.77 ± 4.31	0.069
Age (years old) subgroups	20–29 (%)	52.4%	59.6%	54.2%	0.529
	30–39 (%)	45.1%	38.6%	44.4%	
	≥ 40 (%)	2.4%	1.8%	1.4%	
BMI (kg/m ^2^ )		26.16 ± 4.67	25.50 ± 4.31	26.18 ± 4.82	0.190
Smoking rate (%)		4.5%	6.4%	9.7%	0.110
Duration of infertility (years)		6.42 ± 4.02	6.49 ± 3.18	6.30 ± 3.49	0.410
Etiology of infertility (%)	Male factor	40.2%	41.7%	31.9%	
	Tubal factor	4.3%	1.8%	1.4%	0.069
	Unexplained	35.4%	37.7%	42.5%	
	Poor responder	20.1%	18.8%	24.2%	
Baseline-FSH (IU/mL)		6.94 ± 1.94	7.08 ± 2.19	7.29 ± 2.49	0.103
Baseline-LH (IU/mL)		5.14 ± 2.71	5.48 ± 2.93	5.77 ± 2.97	0.064
Baseline-estradiol (pg/mL)		43.27 ± 13.15	45.62 ± 16.54	44.79 ± 17.36	0.124
Antral follicle count		6.61 ± 2.48	6.63 ± 2.38	6.28 ± 2.58	0.395
TSH (µIU/mL)		2.15 ± 1.04	2.20 ± 1.07	2.19 ± 1.17	0.885
Prolactin (ng/mL)		14.74 ± 7.19	15.62 ± 7.93	15.53 ± 10.20	0.224
Stimulation protocol (%)	Long	24.5%	21.1%	17.1%	
	Antagonist	75.5%	78.9%	80.8%	0.118
Duration of stimulation (days)		10.27 ± 1.42	9.93 ± 1.84	10.13 ± 1.61	0.101
Gonadotropin dose (IU)		2037.62 ± 705.15	1861.35 ± 902.89	1921.41 ± 816.44	0.109
Estradiol levels on day hCG (pg/mL)		2051.67 ± 1110.13	2002.62 ± 1109.04	1859.54 ± 1177.84	0.126
Progesterone levels on day hCG (pg/mL)		0.86 ± 0.38	0.85 ± 0.40	0.79 ± 0.39	0.098
Endometrial thickness on day hCG (mm)		10.56 ± 1.64	10.51 ± 1.61	10.27 ± 1.89	0.130
Endometrial thickness on transfer day (mm)		10.62 ± 1.78	11.01 ± 1.73	10.61 ± 1.90	0.105

Abbreviations: BMI, body mass index; FSH, follicle stimulating hormone; LH, luteinizing hormone; hCG, human chorionic gonadotropine; TSH, thyroid stimulating hormone.

* Statistically significant.

The reproductive outcomes of the participants are summarized in Table 2 . The numbers of oocytes retrieved, MII and 2PN, transferred embryo, fertilization rate, the ET day, the catheter effect and ET technique, and the CPR were comparable between the groups ( *p* > 0.05).

**Table 2:** Laboratory and reproductive outcome parameters of the patients

		Group 1 ( *n* = 164)	Group 2 ( *n* = 233)	Group 3 ( *n* = 360)	*p-value*
Number of oocytes retrieved		9.67 ± 6.59	8.92 ± 4.87	9.16 ± 5.39	0.112
Number of MII oocytes		8.02 ± 4.65	7.30 ± 4.22	7.10 ± 4.38	0.144
2 Pronucleus		5.19 ± 3.43	4.65 ± 3.08	4.86 ± 3.32	0.276
Fertilization rate (%)		63.98 ± 22.30	66.80 ± 23.88	65.35 ± 25.01	0.424
Grade I embryo (%)		64.8%	67.9%	66.7%	0.821
Number of embryos transferred		1.21 ± 0.40	1.18 ± 0.86	1.17 ± 0.38	0.821
Days of embryo transfer (%)	2	12.6%	4.5%	3.1%	
	3	78.0%	83.5%	87.1%	0.193
	5	9.4%	12.1%	9.8%	
Embryo transfer technique (%)	Easy transfer with a soft catheter	21.1%	18.3%	20.8%	
	After external guidance transfer	69.1%	73.3%	70.1%	0.118
	Difficult transfer with a stylet	9.8%	8.4%	9.1%	
The presence of blood in the catheter (%)		2.4%	3.1%	3.6%	0.466
Clinical pregnancy rate (%)		34.8%	37.7%	35.1%	0.771

## Discussion

The present study aimed to investigate whether there is an effect of the physician who transfers the embryos on pregnancy rates in IVF-ICSI treatment. We found no such impact in the present study. There are several studies on this topic with contradictory results; some have shown that the physician factor can have as significant an effect on CPR in IVF-ICSI cycles as clinical and embryological features, 6 14 15 but contrasting results have also been reported. 12 16 17

Hearns-Stokes et al. 17 found that CPR was statistically different, at 17.0 and 54.3%, according to the physician performing the procedure in their evaluation of 854 fresh ETs in 617 IVF-ICSI cycles. However, possible confounding factors such as age and embryo quality, which may have caused this significant difference, were not analyzed. In another study, 1,850 IVF-ICSI cycles were retrospectively evaluated over 2 years, and CPR rates ranged between 13.2 and 37.4% among the physicians who had performed the ETs. When standard training was provided to the physicians with low CPRs in the 1 ^st^ year, the rates increased. 12 However, other factors that could affect the CPR were once more not taken into account in the statistical analysis.

In some Nordic countries, ET is performed by midwives and physicians to share the workload, and in a study comparing 102 ETs split equally among the groups, no difference was found in terms of CPR, at 31.0 and 29.0%, respectively. 18 In a study of 679 nurses and 92 physicians in the United Kingdom, 771 ETs were evaluated and the CPR rate was found to be higher in the nurse group, at 36.2 versus 29.4%. 11 Since the ET procedures in our IVF center were all performed by physicians, these comparisons could not be performed in our study.

In a study evaluating 204 ETs performed by 5 trainee providers, no significant CPR difference was found between them. 13 Since our clinic does not train IVF physicians, no such comparison was performed on our data. Elsewhere, a study comparing the effect on CPR of 2 physicians performing 485 ETs found no difference, at 36.1 and 20.6%. 16 Similarly, in a further study that evaluated 977 ETs performed by 6 physicians, no difference was found between them in terms of CPR. 5

Over the past 10 years, significant efforts have been made to develop standardized and atraumatic ET procedures to minimize the provider effect on CPR. 6 19 In our IVF clinic, for example, a definitive procedure is implemented and performed by experienced and senior physicians; more specifically, USG is used to guide and expel the embryos into the endometrial cavity ∼ 10 mm from the uterine fundus on full bladder. Although ET is always performed by an experienced physician, it should be kept in mind that there are many possible confounding factors that can alter CPR, such as the presence of blood or mucus. These were excluded from the present study. Ultimately, there was no difference between the groups in terms of confounding factors. We subsequently explored the effects of different ET physicians in IVF-ICSI treatment, and found that it has no effect on CPR.

The strong point of the present study consists of its prospective design, the adequate number of subjects in each group, and the prototypical sample from central Turkey. The results can be generalized to most of the population of the country. However, the potential limitations of the study are that it was conducted in a tertiary care institution and that the cumulative CPR was not evaluated because no frozen ETs were included.

## Conclusion

In conclusion, our data show that the pregnancy rates of patients who underwent IVF-ICSI treatment at our clinic were not impacted by the physician factor of who transferred the embryos.
